# Self as an Aesthetic Effect

**DOI:** 10.3389/fpsyg.2019.01433

**Published:** 2019-06-28

**Authors:** Antonia Larrain, Andrés Haye

**Affiliations:** ^1^Facultad de Psicología, Universidad Alberto Hurtado, Santiago, Chile; ^2^Escuela de Psicología, Pontificia Universidad Católica de Chile, Santiago, Chile

**Keywords:** self, dialogical, aesthetics, theory of discourse, affection

## Abstract

Mainstream psychology has assumed a notion of the self that seems to rest on a substantialist notion of the psyche that became predominant despite important critical theories about the self. Although cultural psychology has recognized the diverse, dialogical, historical, narrative, and performative nature of self, as opposed to the idea of self as entity, it is not clear how it accounts for the phenomenological experience of self as a unified image. In this paper, we offer a theoretical contribution to developing the implications of a genetic approach to self in cultural psychology, taking into account an otherwise overlooked dimension: art and aesthetics. We draw on the work of classical authors relevant to cultural psychology, who, although geopolitically and theoretically diverse, are concerted in understanding human psychological life as part of a living process of becoming: James, Mead, Dewey, Vygotsky, Bakhtin, and Vološinov. Overall, the hypothesis developed throughout the paper is that self is produced within psychological individuation as an effect of the aesthetic activity involved in everyday discursive life. We deepen the ideas that self is not an entity but a process of open becoming and that cultural life entails a radical experience of alterity, but we recognize the psychological importance of the sense of unity and closure generated in this process. We argue that self entails not only the process of becoming but also an aesthetical effect of unity in becoming. Self as an aesthetic *effect* emphasizes the self as a discursive and technical process of production, involving a product that, despite not being a finished entity, is felt as unitary and as *mine* by virtue of a specific transformation of experience. We thus propose to define self, on one level, as an epistemological category that points to the paradoxes of identity and agency in psychological individuation, and, on a different level, as a twofold operation that makes possible the subjective experience of a constitutive effort as much as a transient but experienced identity or agency.

## Introduction

τοῦ λόγου δ’ ἐόντος ξυνοῦ ζώουσιν οἱ πολλοὶ ὡς ἰδίαν ἔχοντες φρόνησιν.

(Though the logos is common, the many live as if they had a wisdom of their own.)

Heraclitus

Self is an elusive term (see Strawson, 1997), characterized by reflexivity, agency, and endurance, features extremely difficult to account for (see White, 1999). Mainstream psychology has assumed a notion of the self without much discussion. Since psychology’s inception, this unproblematic notion of self has been criticized for conceiving of self as something, an entity, a finished and self-contained thing: “It has been the tendency of psychology to deal with the self as a more or less isolated and independent element, a sort of entity that could conceivably exist by itself” (Mead, 1934, p. 164). Self as a process, a social process, is then contrasted by Mead with self as an entity. Self-related notions such as self-esteem, self-concept, self-regulation, self-theories, among others, are building blocks of the way mainstream psychology accounts for processes such as motivation, identity, learning, and emotional well-being, among others. Contemporary notions of self in psychology seem to rest on a substantialist notion of the psyche that became predominant despite important critical theories about self that were elaborated with the formation of psychology, by seminal authors as different as William James and Lev Vygotsky. Focusing on the problem of self in particular, we argue that psychological thinking has tended to take the product of the process of becoming as the starting point, attributing substantial reality to it, thus overlooking its dependence over the endless effort of production on which it relies.

In the past, discussion of self has been resumed in cultural psychology from a critical and processual view. Dialogical self-theory (Hermans, 2001), based on the works of William James and Mikhail Bakhtin, has made a critical contribution to theorizing self as a dialogical process of taking different positions. Continuing the thread opened by Ken Gergen in the 1990s (Gergen, 1991) with the idea of a saturated self in contemporary society, Hermans (2001) conceives of self as a multiple and dialogical process of position-taking, but, unlike Gergen’s (1991) view, it is related not to the modern conditions of life but to the inevitable social nature of self. From a sociocultural standpoint, some authors (e.g., Nelson, 2003) have developed a narrative notion of self, namely, self-unfolding through life narratives and autobiographical memory, which brings historical articulation to an otherwise disconnected and fragmentary experience of ourselves. From this perspective, self is constructed. However, in these theories, it is not clear if constructed selves are representations or ontological productions; sometimes they are treated as epiphenomena and sometimes selves are conceived as unities differentiated in part–whole relations or as psychological systems that are the causal grounds of agency. More radical philosophers relevant to cultural psychology, as diverse as Ricoeur (1992) and Butler (2006), have suggested how self involves ongoing narrative and endless performative efforts of constitution as singularities in or through discursive activity.

Although cultural psychology has recognized the diverse, dialogical, historical, narrative, and performative nature of self, as opposed to an assumed idea of self as entity, it is not clear how cultural psychology would account for the phenomenological experience of self as a unified image, or the persistence of substantial conceptions of the self. We think that any persuasive cultural notion of self should account not only for diversity, as its starting point, or its social-discursive constructive nature, but also for why it involves a somewhat unified experience of ourselves. In this paper, we offer a theoretical contribution to developing the implications of a genetic approach to self in cultural psychology, taking into serious account an otherwise overlooked dimension: art and aesthetics. We do so drawing on the work of classical authors who have had a relevant influence in cultural psychology, and who, although geopolitically and theoretically diverse, are concerted in understanding human psychological life as part of a living process of becoming. We refer to James (1890/1952), Vološinov (1929/1986), Dewey (1934), Mead (1934), Vygotsky (1934/1987), and Bakhtin (1952–53/1986a). These authors, from a different era, all faced in their terms (in opposition to formalism, structuralism, associationism, positivism, and Kantianism) the need to develop a theoretical alternative to the philosophies of the substance and the subject, the two predominant models of being, in order to understand experience. Our method was to elaborate a documented interpretation of each author and trace conceptual connections among their theories, in order to mount our argument. The selective exposition of their works, which challenges the ways they have been read within psychology, should be read not as a literature review but as conceptual analysis.

## Self as a Dialogical Effort After Articulation

James and Mead elaborated on their account of the self in dialogue with the notion of personal identity developed in classical empiricism, where *I* is not a given reality of mind but a construction based on perceptual experience, mediated by reflection, habits, and imagination. The theory of self in classical empiricism is already a theory about how “minds” produce identities, so identity is not given. Hume (1748/1952) emphasized that the sense of sameness is not contained in given experience but attributed and fictionalized by the subject through imagination and memory, collecting past experiences and giving them a unity based on the present experience. The radical empiricism of James (1904/1912) implies, on the contrary, that the *feelings* of similarity and difference are part of experience, not added to experience by subjects, and that, as developed by Mead (1934), these ever-new feelings during becoming are never unified to coincide with the present (of “mind”), but generate ever-new gaps or challenges to the building of a potential unity of past and future streams of subjectivity. Thus, with James and Mead, it is not that the subject produces his/her own unity but that familiarities and differences among time-extended and socially distributed thoughts are articulated in the self-individuating production of subjectivity, ultimately yielding the feeling that experience is *mine,* but never the experience that *I* am a simple and complete unity.

James’ (1890/1952) starting point was movement. Thinking, or any form of subjective experience or consciousness, simply goes on, as a constantly changing stream: “*no state once gone can recur and be identical with what it was before”* (James, 1890/1952, p. 149, emphasis in the original). However, the flow is continuous, being part of a *common whole* of subjective life extended in time, that is, a kind of unity at the scale of ontogenesis. Whatever the flow involves, it is *my* flow, which implies that *I* can appropriate, remember, conceive, and feel my past states of thought as mine: the flow is then elaborated in a way that generates in experience a sense of personal self. How is this possible given the continuous change? The stream of thinking is partly organized by an impulse of self-seeking, an impulse for providing the future and not just maintaining the present, which involves dealing with both rival drives persistent from the past and contradictory potential selves or self-projects. The extent to which this self-seeking effort leads to a gap between the actual and the potential (projected or desired) sense of self is related to how this will be felt (self-feeling). The condition of this tendency or impulse of self-seeking is the subjective multiplicity implied by the material stream of experience. In *Principles*, after discussing the production of movement (Chap. XXIII), James argues (in Chap. XXIV, on the concept of “instinct”) that the specificity of human experience is the multiplicity and complexity of impulsive forces compared to other living beings (p. 393), opening the problem of their (im)possible unification.

Furthermore, James defines personal identity, or the “personal form” of experience, as “the sense of sameness perceived *by* thought and predicated of things *thought-about*” (James, 1890/1952, p. 214, emphasis in the original). The self, as a process and a relation, implies a constitutive difference between *me*, the perspective of everything I can call mine, such as my body, past experiences, and singularities, and *I*, the perspective of the subjective position from which certain tracts of experience are felt as mine or not, evaluated and thought about. This means that self implies both objectification and subjectification, because it involves the synthesis of the agent (*I*) and object of thought (*me*), which is not a dialectical synthesis, because each act of appropriation of oneself generates a new *I* position that is not contained in the experience of *me* that is integrated gradually at each moment. Thus, self is not a state, thing, or entity (see Zhao, 2014), but a reflexive operation of (self) differentiation, a struggle toward unity by means of the ever-new introduction of a constitutive difference. The subject, according to this theory, cannot be totally self-appropriated because each subjective act of appropriation is only provisional and engenders a tendency toward new potential paths in the self-production of subjectivity that are not contained in past experience. In this sense, we may be allowed to call this, following Bakhtin’s discussion about this distinction in his late work (Bakhtin, 1974/1986b), not a dialectical but a dialogical theory that conceives the self in opposition to the modern notion of the subject as a unitary and self-determined center of agency.

We draw some specific implications of James’ interpretation from the idea of self as *tendency*. Despite the fact that we have the experience of unity and closure, self is not the successful production of this unity, but a constant partially unsuccessful operation of returning over our past states of mind, which in itself produces novelty and difference. Self, as a microgenetic operation and ontogenetic activity, is always incomplete and only effective as a productive tendency. It is a problem, a challenge, addressed with diverse strength at different moments and in different individuating drifts. More than a reality or a fact, the unity of self is a promise to others and oneself that can only be pursued through self-differentiation (a promise we can neither keep nor abandon). Self refers to a psychological work within individuation, a process-product, and not to an individual reality or end-product of individuation. As such, self involves a temporal feeling of unity implied in the directionality of its promise, where the felt unity is taken as neither a fact nor a cause but as an emergent tendency, an *effect*. From here, we take one sense of the notion that self is an effect, according to which the problem of self calls for a theory about the performative work that has consequences in becoming, as James understood his “pragmatism” (James, 1907/1955).

Following James, Mead (1934) elaborated the idea of self as a socially mediated process of articulation of *I* and *me*, giving a clearer role to language and discursive communication. The key idea is that self arises through social and linguistic interaction because it is through this, through the perspective of others, that it is possible to become an object to oneself. For Mead, this means not to become a thing but to develop an *experience* of oneself, to *take a perspective* or *attitude* toward oneself. Self is not a mental construction but a “structural” process, a social process, in which the other’s perspective toward my action (also a perspective) is appropriated or internalized, so that it can be taken by me toward my own actions. These perspectives that we take to ourselves are not intellectual, which is why he refers to them as attitudes: they are emotional and axiological positionings regarding other ones. Language is crucial because it is a conventional medium in which perspectives are not only taken but also shared and recognized by different people who are affected in similar ways by their expressions, just as these expressions, qua utterances, expect somebody else to be affected.

Mead also recognizes the diversity of subjectivity in terms of how different perspectives emerge in different moments of social life: “We are one thing to one man and another thing to another. (…) We divide ourselves up in all sorts of different selves with reference to our acquaintances” (Mead, 1934, p. 142). However, self means not just a relational process in which two parties are involved (*I* and *Me*). The reorganization and articulation (or integration, according to Gillespie, 2005) of this diversity are reached not through subjective synthesis, but by reference to the community to which he/she belongs because we do not just internalize specific perspectives of ourselves communicated to us by others; by appropriating them, we also appropriate social norms that organize our communal and institutional life, thus appropriating a “generalized other.” It is through the ontogenetic internalization of a generalized other that social groups influence and exercise control over the behavior of individuals by entering it as a determining factor of their own thinking.

According to Mead (1934), the feeling of unity is an abstraction because, on the one hand, what unifies the experience is the social organization of self that involves different perspectives and attitudes in particular relationships. On the other hand, self depends on the performance of other selves, other’s self-unification works and projects. Individuality consists precisely in the singular *importation* into subjective life of the broader ideological and axiological organization of the community, realized as intersubjective or group tendencies in concrete social interactions. Moreover, the singular appropriation or articulation of social normativity, as an individual self-unification work and project, must be recognized by the community as a member of a collective individuation context. Selves are social processes that are realized singularly in each case through others’ perspective. Self is not a self-contained unity; according to Mead, it needs to be constantly completed by the recognition of others, and it is always awaiting that recognition.

The paradox of self is that experience, within self-individuation processes, is always demanded to realize a unitary organization of feeling and thought around the agency of the *I* but condemned to search and strive for it through others. A unitary organization based on the sameness of the subject is theoretically impossible. Even on a microgenetic scale, unification never takes place completely, insofar as there are many selves or self-projects struggling for unification interdependently and with frequently conflicting goals derived from the conflicting social interests within society.

## Self as a Dialogical Operation in Discourse

On the basis of our interpretations of James and Mead, we follow Bakhtin’s notion of language and discourse to define four features of self as an operation:

*Dialogic*. Self is a reflexive operation that has a social structure. Which structure is that? The structure of alterity: the response. Through the use and internalization of language practices, we acquire the capacity to objectify ourselves, to take perspectives, to respond, to ourselves. Because of this dialogical structure, we become able to have an experience of oneself. We conceive experience in the sense that Mead does: to have an experience is to take a perspective of, to respond to.

*Normative.* Insofar as the object-subject dynamic is referred to the generalized other (i.e., the normative aspect of self), self is a dialogical operation with a tripartite social structure: perspectives addressed to one another (I/We and You/They) and a generalized voice (Society). This theoretical scheme in Mead resonates with another aspect of Bakhtin, the figure of the *third* or *super-addressee* (Bakhtin, 1959–61/1986c).

*Performative*. Self is not only an activity rather than an entity, with some preferring to talk of *selving* instead of self (Thibault, 2019), but a dialogical operation that must be constantly performed in direct dependence of other selves. Self operates not only to articulate difference, but to do it in a particular way that can be recognized by others, according to the community norms. However, an identity resolution pursued through others’ recognition, which mediates experience beforehand, never arrives because this social mediation renders self-individuation open to ever-new forms. Therefore, it is a dialogical operation in its own right: it is opened and inconclusive.

*Discursive*. Self unfolds through, and as, discourse. For this reason, as we read Mead (1934), it is dialogical, normative, and performative. From our reading of Bakhtin (1952–53/1986a) and Vološinov (1929/1986), discourse is a process of ideological engagement unfolding through different and juxtaposed languages. Discourse is the tensioned and dynamic field in which different perspectives, angles, voices, interests, and worldviews emerge through the materiality of words and languages in relations of contestation, opposition, agreement, and neglect, among others, many of which are in the same stream of discourse. As such, discourse is the process of human communication that involves different semiotic means (verbal and non-verbal) through which meaning is dialogically and dynamically produced and transformed, in social and individual realms. Thus, discourse is not something that happens *outside* or *between* individuals but a process that goes through them, questioning the boundaries between the social and the individual, the inner and the outer, the mine and the others, and constituting them from the inside (Vološinov, 1926/1976). Discourse and language do not represent realities, as duplicating worlds, but assume the material conditions of life as integral and constitutive parts of the process, through which a univocal view of the world is transformed into a perspectival, relative, engaging, historical view, inscribed in social and axiological hierarchies. Therefore, saying that self is discursive is to say that self is a dialogical operation that unfolds as the articulation of ever-changing and partially shared worldviews materialized in different social language uses, which has constant affective consequences. Self unfolds in the boundaries between mine and yours, outside and inside, the assumed and the explicit, among others.

However, self is not only a discursive dialogical operation; it involves the experience of unity. We do not appear to ourselves as ever-changing and disaggregated parts. We are not aware of the failure of resolving ourselves. How can we explain this? It is reasonable to accept that self-organizing systems can generate and recognize changes in their environment that in the long term inform their agency, so that the emergence of self-consciousness from computing mechanisms is expected [as demonstrated again by Friston (2018)], if not necessary [as suggested by Bergson (1896/1912)]. However, the question is how, in what forms, and with which subjective and objective consequences, real human beings deal with the problem of the multiplicity of experience beyond adaptation to the present. In our argument, human beings are considered not specifically as self-organizing systems but as socially mediated processes of self-individuation. The work of self takes place as long as we are participants of different cultures involving routines and repetitive social practices, shared beliefs and values, rules and norms, and institutions (see Zittoun, 2008). The repetitive encounter with others, playing specific and delimited roles in activities framed by shared norms and values, and the ongoing process of remembering, contribute to the feeling of sameness or enduring through experience. Regarding rituals and practices, the stability of these social practices contributes to one’s recognition of the one that was yesterday, and its rituality provides relevant resources for identity formation.

This repetitive aspect of social practices is embodied in typical forms of utterance that Bakhtin (1952–53/1986a) conceived of as speech genres, only within which or in relation to which every utterance is unique and unrepeatable. In this sense, discourse involves difference and uniqueness but also repetition and stability. The typical forms of using language provide relevant information about presuppositions, offering resources to recognize speakers and addresses in their intentions and types. In this sense, cultural life involves norms as genres of behavior that frame social activity and condition the possibility of social recognition (see Butler, 2006). This normativity of social behavior is also a product or effect of discursive life. Norms and models are based on evaluative and axiological structures constructed historically and enacted through the use of language, operating as implicit or explicit voices in discourse. As norms endure and are typically widely shared in a given culture, and as subjects emerge, necessarily establishing dialogical relations of subjection, contestation, or both, they also offer important resources to the idea of subjective stability and endurance.

Nevertheless, practices and norms are not univocal, so they cannot unify experience. Moreover, through their tendency to establish a shared cultural world, they also conflict and tension subjectivity, imposing a power structure within the flow of co-affection, and diversifying and breaking it at the same time as they try to hold it through time. This is particularly the case because we live not in one homogeneous culture, but rather in intersections and ever-changing boundaries between different cultures and norms. In Bakhtin’s (1934–35/1981) words, languages sediment different value and normative systems, but no speaker speaks only one language. Each word is the intersection between different languages that have used the same word for different purposes and in different axiological systems. So, when speakers use a word, they stand in a dialogical and intersectional territory in which different and contradictory norms demand their own responses. Therefore, routines and normativity, as much as they stabilize and homogenize, diversify and tension the flow of subjectivity. So, again, how is it possible to account for our provisional feeling of unity, based on which a whole psychological theory of subjectivity as an individual substance has been constructed?

Another relevant dimension is memory and history. As Katherine Nelson (1993, 2003) argued, following Ricoeur and Mead’s ideas, self develops intimately associated with autobiographical memories and narratives (see also Wang and Brockmeier, 2002; Fivush et al., 2011). The personal sense of self is less about sameness than about inscribing oneself in shared narratives through which we get a historical structure and, in turn, a sense of continuity and singularity in relation to immediate and broader alterity. The implication of this way of thinking about self is that language, through its narrative potential, allows articulation of oneself historically in relation to a broader social context, so that the feeling of unity is a product of historical memory that articulates and knits not only different episodes of one’s own life but also our life to social broader episodes, giving them density and inscribing them with a meaningful process of becoming.

However, stories about our life are not logical narratives but fiction, which involves an artistic and aesthetic dimension that may contribute to the kind of experience that the operation of self involves. The role that the artistic and aesthetic dimensions of narratives may play in the achievement of this sense or feeling of self has mostly been overlooked in cultural theories of self. Much attention has been paid to the role that memory and history play in the development of self, both of which are extremely important but which overlook the role that less rational dimensions, such as imagination and emotion, may play in it.

## The Artistic Work of Self

### Taking a Unified Perspective of Oneself Through Art

John Dewey, in one of his late works, *Art and Experience* (Dewey, 1934), argues for the artistic and aesthetic dimension of everyday life. He reflects on the aesthetic underpinnings of the process of objectivation of the flow of experience, suggesting a strong link between self as a work of unification of experience, and art as a work of imagination toward transforming emotional and bodily experience. He critically discussed the idea of art as a specific and isolated realm of culture, and aesthetic experience as different from common, everyday experience. In short, art, as an everyday life activity, facilitates the objectification of experience, the transformation of a flow of experience into something we can have an aesthetic experience of, through its emotional unification.

According to Dewey (1934), experience is transit, movement, experience-in-becoming, in which emotions play a key role. Emotions are not an individual matter; they are socially shared and intimately intertwined with meanings and shared values and beliefs. As part of experience, emotions go on. We are typically engaged in experience as an affecting flow that goes on. Experience also involves a reflective dimension. We can intellectualize aspects of that experience, being able to think about it deliberatively; for instance, in the case of scientific thinking through which we imaginatively hypothesize and elaborate plans of intellectual action to reach conclusions. However, there are aspects of the flow that cannot be intellectualized, remaining desegregated pieces (blind spots) of the flow of experience but maintaining affective productivity; for instance, aspects hidden in the depths of our personalities as a building block thereof, or which are part of our memories or anticipations in a fuzzy way. Art, as an expressive activity in which objective and shared values are enhanced, calls for specific emotions, promoting the articulation of the otherwise disarticulated aspects or parts of experience that are felt in the same way. Art, therefore, through its expressive character, helps the integration and articulation of experience, giving it aesthetic density.

Art is not only a differentiated sphere of culture but it operates in the microgenesis of everyday life; but experience as a flow is not necessarily aesthetic, becoming aesthetic through artistic expression. Emotions, however, are more than simply gathered by artistic activity; they are transformed and imaginatively created as an object of experience, becoming attached to the object produced by the artistic expression. Artistic expression has a productive power over experience, transforming the flow into a totality, an object toward which we can take a perspective, a response. It is important to note that we are using experience in two senses: experience as the flow of experience in which we live and having an experience, or taking a perspective (following Mead, 1934). The aesthetic dimension of experience is the artistic affective integration of lived and imagined aspects of the experience. However, this integration does not represent the dissolution of tension or differences (as inferred from Giordano, 2017). It could be precisely the unification of contradictory emotions in one and the same stream of life.

Vygotsky (1925/1971) recognizes as the main potentiality of art the technical juxtaposition between different and contradictory emotions to produce an affective reorganization of experience. From Vygotsky’s work (for example, *On Psychological Systems*, Vygotsky, 1930/1997) one may read that human emotions are culturally created and transformed through the use of language. In other words, the invention of language transformed not only thinking, memory, attention, among others, but also emotions and imagination. Moreover, he developed the idea that the whole personality is historically transformed and mediated by a specific system of concepts that every individual socially develops through social life. People living in different cultures do not feel the same way: we culturally and discursively create singular emotions and affective responses. In this sense, not only is affective life part of the creative effort of becoming, but this creative process is done through social techniques such as language use. This has different implications. First, emotions and affections are not given but have a historical and arbitrary nature. Second, they are not something that happens to us (passions) but something that we create, recreate, and alter technically, through the use of language. Third, emotions are not individual matters but collaborative: we feel and are emotionally affected like others and because someone taught us to do so.

Vygotsky conceives of art as a social technique for altering emotions. Although in social life, emotions are always a social and technical creation; in artistic expression, this is enhanced. It is as if art were the *technique of techniques*, that is, the manipulation of the otherwise conventionally used material form (signs) to create a completely unique emotional experience. This experience is characterized on the one hand, by the possibility of juxtaposing two contradictory emotions (catharsis), but also by the embodiment and objectification of shared and assumed emotions, meanings and aspects of social life, which, experienced as such, produce new emotions (see *On the problem of the psychology of the actor’s creative work* (Vygotsky, 1936/1999).

Dewey and Vygotski pointed out five relevant aspects of art and aesthetics: (1) art is not an isolated cultural realm but part of everyday life; (2) it has an essential affective and emotional productivity; (3) it involves the totalization, unification, and objectification of experience in the sense that we can have an experience (take a perspective) of an otherwise elusive flow; (4) it is a technique through which we can enhance and create emotions by artificially juxtaposing even contradictory ones; and (5) art is part of language activity and verbal creation; or, in other words, it unfolds through discourse and develops only within its historic, dialogic, and semiotic possibilities, even when no words are involved.

### Self as Author and Hero in Verbal and Everyday Art

Life, discourse, and art are not taken as the same. Although art may be conceived as an intrinsic dimension of everyday life, there are some general notes to make about the artistic import to life from a dialogical theory, before we apply them to the self.

### Technical Fabrication

Since ancient times, art has referred critically to the technical nature of social life and, as such, it is opposed to what is given. Art involves a more or less voluntarily fabrication through which the given is transformed arbitrarily. In that sense, it allows people to contest what appears at some point to be irremediable conditions of life, at both a social and a personal level (death, illness, personal biographies, love deployment, macro-political events, macro-economic structure, among many others). Although one may say that life and art are two different domains in culture, authors such as Bakhtin (1924/1990a) and Vološinov (1926/1976) argue for their intertwinement: life becomes aesthetized and hybrid, so it is then very difficult to distinguish clearly between art and life.

### Objectification and Unification

As dialogic contestation, art is technical, not because it transforms matter (the matter of stone, canvas, scene, bodies, words, etc.), but because it refers to objective values; it is directed *axiologically* toward them (see Bakhtin, 1924/1990a). Moreover, artistic production involves the technical fabrication of a unity, an individualized and objectified object of an otherwise diverse stream of social life. Artistic productions, therefore, involve, through the manipulation of matter, the production of aesthetic objects; that is, transformation of the complex streams of chaotic and dialogical, meaningful and axiological relations in which we are co-participants, in objects of reflection, contemplation, and analysis: “Form, embracing content *from outside, externalizes it, that is, embodies it”* (Bakhtin, 1924/1990a, p. 221, emphasis in the original). Thus, art, insofar as it externalizes, allows subjects to contemplate as objects parts of their own stream of social life (see Cupchik, 2002), which Bakhtin refers to as extra-position, outside-situatedness (Bakhtin, 1924/1990a) or *outsideness* (Bakhtin, 1920–23/1990b). Through this process, intimate and assumed worldviews are made strange, rendering them more clearly visible against moral, axiological, and ideological backgrounds. This general statement of Bakhtin’s theory of discourse is confronted from his early works to the problem of the self, for instance, discussing how autobiographical genres deal with the impossibility of closing or finishing oneself, with the radical difference between author and hero, in terms of what we may call the discursive techniques of alterity.

### Art as Actively Appropriated

It is particularly interesting, and useful in terms of what we are considering, that Bakhtin (1924/1990a) erases the boundaries between artist and spectator (see also Sundararajan and Raina, 2016). To contemplate is not to have an external cognition of the aesthetic object, but rather to become an author, appropriating that object and responding to it; that is, establishing dialogical relations with this new whole that would otherwise continue as a stream.

### Juxtaposition of Alterity

Aesthetical experience, from this point of view, is related to form and its sensuality: dynamic events appear to us as objects because a given relation between form and matter refracts and redirects in specific ways to concrete shared and assumed axiological and ideological horizons. The sensual property of a given aesthetic object, however, is not self-sufficient; rather, it depends upon historical and previous connections to meaningful life that form/matter used to have in a given social group. Aesthetical objects, through their sensual properties, refract, and redirect in new, often unconventional and contradictory, ways, as they typically mix in arbitrary and creative ways forms originally referred to as different spheres of axiological life.

### Inner Dialogicality

Although form is a crucial aspect of language activity (see Bertau, 2011, on “phenomenality” of language), the particular artistic and aesthetic dimension of language is not its sensuality, but the fact that in the use of language, and in some more than others, different language uses, voices, perspectives, and so forth, are artificially and simultaneously juxtaposed, representing aesthetically the multiplicity of discursive life, accentuating ideological and axiological tension, and objectifying dialogicality in order to be experienced as such (see Bakhtin, 1934–35/1981). What ensues is that neither is every use of language artistic, nor is art something that involves only professional artists (see Glǎveanu, 2011 and Tateo, 2017): as we use different speech genres to communicate with one another and to relate to ourselves (see Larrain and Haye, 2012), we often, without noticing and on a daily basis, use artistic utterances to deal with relevant aspects of social life that benefit from the production of aesthetic objects out of the stream of discourse and social life, and estrange ourselves from them. Every one of us organizes our life at some point, whether playing and/or listening to music, writing and/or reading stories, novels or poems, watching movies and series, and painting or enjoying exhibitions, theatre or dance performances, among others. These cultural practices cross our daily lives, penetrating them so deeply (see Bakhtin, 1924/1990a) that they also penetrate, transform, and constitute our psychological functioning from within. We engage in artistic or aesthetic modes of being, not just as individuals interacting with other individuals, but also as a mode of relating with ourselves.

## Everyday Aesthetical Practices of the Self

Self, as something that is felt as unitary, can be conceived of as an aesthetic effect; self refers to the emotionally unified experience through the creation of an aesthetic object. This is not to argue that self is an illusion (Hood, 2012); rather, it is a constant effect of the artistic dimension of discourse, through outer and inner speech. It endures as a feeling of unity because of the repetitive and normative character of social life, which must be constantly performed. Effect here is used following James as an emergent tendency. It does not mean that self is causally produced as something, as an entity, but that the feeling of unity is an emergent tendency that is aesthetic in nature: we experience ourselves as aesthetic objects, as consummated unities; we experience our dialogicality responding to it as an aesthetic whole. The artistic manipulation of form/matter through which the dialogicality of life is immersed, we presuppose, is technically and arbitrarily presented in accentuated tension as an object outside us, as a *strange* that we can contemplate and appropriate. By doing so, we are emotionally engaged in new ways but as a unified and emotionally resolved active contemplator. Artistic expression creates its public in the sense that it emotionally unifies it, resolving (not dissolving) the diversity of its multilayered and dynamic experience.

For instance, when accounting of ourselves explicitly by telling our life stories in therapy, when we first meet a friend or lover, or when we are troubled, trying to understand something about our lives, we construct not just our history, articulating our past and present (memory) with our desired futures (imagination), but ourselves as characters, as aesthetic objects. By doing so, we make ourselves objects of our experience (duplicating ourselves in author and hero), and doing it, so that others and ourselves can contemplate us in a given emotional way. We are in relative control of the story and intentionally we can pick up aspects of our life and juxtapose them with others that are arbitrarily picked up and that changes according to our interlocutors, social situations, and goals. However, we do this to produce emotions not just in others but mostly in ourselves. Often the consequence of this storytelling is relief and sympathy. Relief is because we confirm that we are not given, but we can choose the literature in which we want to live; that we can fictionally and imaginatively repair our past and project our futures in a playful way, which has this concrete emotional consequence. Sympathy is because we can engage with ourselves, experience ourselves, take an attitude of understanding to ourselves, and respond to ourselves in a sympathetic way. By telling our life stories we can transform the way we feel about ourselves. Again, this artistic everyday life activity plays a crucial role when falling in love: passing long hours talking about ourselves involves discovering new ways in which we can experience ourselves as characters, and through the appreciation and interest of others in them, we can appreciate ourselves too. Something similar happens in some therapeutic interactions: we are guided to tell our life stories in such a way that we distance from and experience ourselves in generative and often contradictory ways, that is, aesthetically. For instance, at some points, we need to acknowledge painful and dark aspects of ourselves, which we are ashamed of, but we do it with a sympathetic and forgiving attitude.

We tell life stories frequently, but not always; indeed, this is not the only way in which we create ourselves as aesthetic objects. We also dramatize many situations in our life, positing ourselves as characters in an ongoing dramatic script, accentuating and, again, juxtaposing different pieces of our discursive life to make something highly visible, or creating a version of ourselves that others and ourselves can resonate with. These dramatizations, inscribed in other types of discursive activity and hybridized with them, unfold through not only verbal signs but also motor actions, gestures, and relations with the material scene, among others, which refract meaning in complex ways and are used to perform the drama.

We stylize our bodies through clothing and fashion, through technical interventions such as piercings, tattoos, and aesthetic surgeries, among others. We decorate our intimate spaces. Although most of the time these are non-verbal actions, they are meaningful because they refer to shared values and norms and imply positions toward them. We do it as a way to identify (see Watzlawik, 2014) and diversify from others. These are artistic productions that have the aesthetic effect of creating self. We intentionally choose diverse form/matter relationships that combine and juxtapose in new and unique ways, as a way of choosing our own literature (distancing or attaching ourselves to the one given), thus arbitrarily and intentionally producing a unified (not necessarily homogeneous) emotional experience and the feeling of self. Arguably, this is also a way to struggle with the open possibility of social rejection, repudiation, and indifference.

This is not to say that we write full autobiographies or perform full theater plays. Micro-dramatizations, micro-storytelling, and micro-stylizations are embedded in social life in a responsive dialogue through which self achieves outsideness, taking a perspective toward its blind spots and desegregated parts, while art takes relevance and seriousness from life (see Bandlamudi, 2015). Therefore, this feeling of self, as an aesthetic effect, does not endure unless we are part of routines and normative social experience in which we can recreate in new and different ways these artistic actions. The enduring feeling of self, as something that constantly changes in its emotional tonality and content, responding to the dynamic, diverse, and fragmented character of alterity, needs constant artistic activity and endless performing.

Self as an aesthetic effect involves both an ontogenetic and microgenetic dimension. It involves a microgenetic effort of creative genesis of an experience of ourselves that we have developed so far. But it can also be viewed ontogenetically. The continuous micro-work of self-creation has products (feelings of unity emotionally tonalized) over which self-creation operates. This is to say that self is historical because past creative work impacts future work. For human beings, subjectivity is plural but the microgenesis of experience involves the ontogenetic experience of an effort and a tendency after unity; this work of self is mediated by the incorporation of otherness and normativity, so that self-experience emerges from social experience; and only through self-objectivation can human beings develop enduring cultural identities, agency and a sense of personhood. Moreover, this work unfolds embedded in social practices and institutional demands that change over life. So, self as an aesthetic effect may vary in its intensity depending on life circumstances. For instance, regarding life story-telling, dramatizations, and stylizations, it is possible to think that they are intensified in transitional and liminal periods, where social and institutional conditions of life change (see Zittoun, 2008; Zittoun and Gillespie, 2015; Stenner, 2018). For instance, it is likely that adolescence and old age are periods of life in which life story-telling happens more often: in the case of the former, to distance themselves from parents and families’ stories and literature, choosing their own one; and in the case of the latter, as a struggle with the disintegration derived from the progressive loss of the typical activities performed during most of life, and the death of friends and peers and, with them, the sharedness that holds selves tight.

## Discussion

### Conclusions

Overall, the hypothesis developed throughout the paper is that self is produced within psychological individuation (in the sense of Simondon, 1989) as an effect of the aesthetic activity involved in everyday discursive life. We take as our starting points the idea that self is not an entity but a process of open becoming and that cultural life entails a radical experience of alterity, but we recognize the psychological importance of the sense of unity and closure generated in this process and give a critical account of the sense in which we are something that we can esteem, understand, have theories of, regulate, and so on. Self as an aesthetic *effect* emphasizes self as a cultural and technical process of production, involving a product that, although not a finished substance or entity, is felt as unitary and *mine* by virtue of a specific transformation of experience that should not be taken for granted, or as natural. The aesthetic dimension of experience, associated as it is with the creation of new forms and the production of objects as finished wholes, could be a key piece in the effort to account for self. Self entails not only the process of becoming (dialogical, normative, performative, discursive, and artistic) but also an aesthetical effect of unity in becoming. The effect of self in experience must be regarded as neither a passing feeling nor an isolated microgenetic operation (e.g., the representation by the author of an utterance within the utterance) but as an ontogenetic activity that is a real continuity of experience with varying degrees of selfhood production at different moments, even if unconscious and not represented at any given point. The individuation of living-speaking beings is a social process that has, along its unfolding, real consequences in the subjective organization of experience. In this context, our contention is closer to biosemiotics (see Thibault, 2017) than constructionism (Gergen, 1991). The work of self is not just an invention of modern subjectivity (c.f. Taylor, 1989) but a culturally diverse aspect of experience marked, we argue, by outsideness and the temporal density of becoming. The feeling of being self is not univocal but *singular*: possibly in an infinite number of forms the ontogenesis is constantly resumed, reappropriated, and transformed in microgenesis, forms that are sensible and which, at some points, give a strong feeling of this effort.

Self is an elusive concept because its reference is not an entity, the reflexive individuality of subjective experience, but a problem to be addressed without possible resolution. As such, we may define self, on one level, as an epistemological category that points to the paradoxes of identity in psychological individuation, and, on a different level, as a twofold subjective experience of a constitutive effort as much as an experienced (failure of) identity.

### Implications

We have argued for a conception of self as a dialogical operation in which diverse positionings, ideas, perspectives toward different and contradictory aspects of social life, are effortfully and endlessly articulated. In doing so, we see self in a dialogical way that is different to that first proposed by Hermans et al. (1992) and developed by Hermans and colleagues. According to them, self is “a dynamic multiplicity of relatively autonomous I positions in an imaginal landscape” (p. 23). The idea is that these I positions move from one position to another, endowing them with voices that establish dialogical relations (dialogues between characters) with a narrative structure. Dialogical here is understood as the narrative dialogue through which different characters, or imagined and provisional positions, change information regarding their worldviews. We, on the contrary, take the idea that self-dialogicality consists not in the dialogue between I positions, but in its responsive structure: self is dialogical because it involves the operation of taking oneself as an object of experience, something that is enabled by the structure of alterity. However, self is also dialogical because the structure of alterity involves two parties and a third that acts as a point of reference toward which any experience of oneself becomes a proper experience: an attitude, an emotional stance toward oneself, given an axiological socially shared horizon. Finally, self is dialogical because it is constantly performed in social interdependence, addressed to others’ recognition and rendering self-closure never achieved. Moreover, although Hermans et al. (1992) give a clear role to imagination in dialogical self-theory, it is mostly in the form of imagined characters and *I* positions. Self as an aesthetic effect involves imagination as an active process of ongoing creation, in which virtual aspects of experience take a central role, even when they are not always represented.

Contrary to the available perspectives (see Brown, 2001), and in accordance with Tateo (2017), we have argued for the artistic dimension of everyday discursive life, discussing the separation between discourse and art, the latter traditionally understood as a separate and isolated sphere of cultural activity. This is a conflicting and debatable position, particularly because art is related to aesthetic values: not every artistic production is a work of art. However, art is not the same as a work of art, and the former should not be defined by the latter. Artistic productions may have different cultural values, but this does not remove them from art as a practice. We define art as the technical and thus an arbitrary and culturally variable fabrication of an aesthetic object that involves the intentional juxtaposition of different worldviews, independent of the aesthetic value of that object. In this sense of the term, art is part of everyday life. We sustain the idea that the doing of self is a kind of art, although not in the same way that plastic or performative arts participate in aesthetic production. We contend that subjects are part of the self-production of experience within social individuation drifts, a self-production that is not a subject or a self in the order of beings but a creative process in the order of becoming.

One limitation of this initial sketch of a theory of self is the scarce consideration given to power, something that has been identified as difficult using Bakhtin’s theory in cultural psychology (Sullivan, 2007), which should take into account power and normativity when theorizing about subjective life. In this case, the historical organization and validation of some subjects over others, which operate through normativity, frame the artistic possibilities of a given participant in discursive life. They therefore determine the aesthetic and emotional content of a given self. However, this determination is never complete, precisely because normativity is not monological and divested of inner dialogicality. In this context, power is related not just to the determination, limitation, or subjection of the subject but more radically to the technical implications of aesthetic activity to understand the political aspects of self.

Further research may show that an ontogenetic and dialogical theory of self is better prepared to account for transformations in normative and subjective constituents of experience, compared to theories of self as system or autonomous agency, for instance, showing the role of aesthetics and power in becoming gendered, classed, or racialized.

## Author Contributions

AL co-led the research, drafted the first version of the manuscript, and reviewed the final one. AH co-led the research and drafted the second version of the manuscript.

### Conflict of Interest Statement

The authors declare that the research was conducted in the absence of any commercial or financial relationships that could be construed as a potential conflict of interest.
